# Resource Utilization and Emergency Medicine Advisors’ Approach to Video Interview Preparation

**DOI:** 10.7759/cureus.18504

**Published:** 2021-10-05

**Authors:** Mark Olaf, Shannon Moffett, Matthew Ledford, Megan Fix, Liza Smith

**Affiliations:** 1 Emergency Medicine, Geisinger Commonwealth School of Medicine, Scranton, USA; 2 Emergency Medicine, Rutgers University, New Brunswick, USA; 3 Emergency Medicine, University of Connecticut School of Medicine, Farmington, USA; 4 Emergency Medicine, University of Utah School of Medicine, Salt Lake City, USA; 5 Emergency Medicine, Baystate Medical Center, Springfield, USA

**Keywords:** standard video interview, residency application, advising, application preparation, emergency medicine

## Abstract

Introduction

The Standardized Video Interview (SVI) was a residency application component introduced by the Association of American Medical Colleges (AAMC) as a supplement to the existing process, which aimed to measure knowledge of professional behaviors and interpersonal skills. Given its novelty in both aim and execution, little advice or experience was available to inform preparation strategies. We sought to perform a cross-sectional analysis to explore advisors’ practices in guiding students’ preparation for the SVI.

Methods

An electronic questionnaire was developed and piloted for flow and usability, then distributed to all members of the Council of Residency Directors in Emergency Medicine (CORD EM), the professional society for emergency medicine educators, via listserv, comprised of 270 residency programs. Questions were both open- and closed-ended and therefore analyzed in a mixed-method fashion.

Results

We received 56 responses from a listserv representing 270 residency programs. Respondents cited personal experience and consensus opinions from national organizations as the primary sources for their advice. The most common resources offered to students were space for completing the SVI (41%) or technical support for completing the SVI (47%). The time committed to student advising specifically for the SVI ranged from zero to 20 hours. Estimated associated costs of preparation ranged from zero up to $10,000 (time plus resources). Two individuals reported recommending commercial preparation resources to students.

Conclusion

The SVI was a novel attempt to augment the resident application process. We found variability in resources and advice offered to students, including broad ranges of time dedicated, the monetary value of resources contributed, and the types of resources utilized. As the global COVID-19 pandemic has inspired a wave of innovation and process changes, we present this data for consideration as a snapshot of the variable responses to a single uniform process change.

## Introduction

The Standardized Video Interview (SVI) was a novel residency application component introduced by the Association of American Medical Colleges (AAMC) and piloted with the Emergency Medicine (EM) Residency application process, with programs receiving results of the score calculated in a reportedly standardized fashion by an independent body. The SVI was created in response to a perceived lack of reliable methods for assessing an applicant’s interpersonal communication skills and professionalism as part of a holistic application review. This gap was identified based on the results of the 2016 Program Directors Survey [1].

The format of the SVI utilized an online, unidirectional, video-recorded interview. Students were presented via an online interface with a total of six written questions. For each question, 30 seconds were allotted for the applicant to read the prompt and reflect, followed by up to three minutes to record the response via the interface. Students did not have an opportunity to pause or delay the response. The exercise attempted to assess the Accreditation Council for Graduate Medical Education (ACGME) competencies of interpersonal communication skills and knowledge of professional behaviors and add a unique facet to the residency application portfolio. Responses were scored on a scale of 1-5 by standardized AAMC-affiliated reviewers, using an algorithm that was not shared. Analysis of initial SVI results showed little or no correlation with NBME Step 1, Step 2 Clinical Knowledge (CK), or Step 2 Clinical Skills (CS) testing [2-4]. 

The AAMC offered advice to help students prepare for the SVI via a preparation guide. Recommendations for preparation estimated by the AAMC to take 1-4 hours included: reading the guide, completing one or more practice interviews on the online interview platform, studying sample questions, and rehearsing responses without and with technology [5].

EM-bound students have been queried about self-study practices for preparing for the SVI [5]. Multiple authors have examined the SVI scores and their correlation with the likelihood to interview [6], faculty and patient ratings [7], and interview performance [8]. Student reactions to the SVI were generally negative [9] and SVI scores did not significantly correlate with specific preparation strategies [10]. Bird et al. suggested that further research look into preparatory practices for the SVI [2]. We sought to evaluate the advisor perspective and resources allocated to this novel process.

## Materials and methods

Our study received multi-institution IRB approval. We generated an exploratory questionnaire (appendix 1) based on suspected gaps in knowledge, after a literature review of resource utilization for preparation for the SVI. The instrument was developed by one author (MO), then reviewed in an iterative fashion by the other authors, all of whom were experienced advisors and involved in advising EM students regarding the SVI. Our questions aimed to identify the role of the advisor, the number of students advised, the source of advice, and specific details regarding SVI advising and preparation resources. Question format included multiple-choice and open-ended questions. We then surveyed emergency medicine educators through the Council of Residency Directors in EM (CORD EM) listserv that comprises 270 residency programs and affiliated medical schools. Survey data were collected in Qualtrics from August 13, 2019 to September 1, 2019, after the window for that academic year’s SVI had been completed. The data were reviewed manually by two reviewers (MO and SM). Quantitative data were aggregated and reported, while qualitative data were categorized by theme. Blank responses were excluded from data analysis. There was no tracking to preclude multiple responses from a single individual, nor were data provided verified independently.

## Results

**Figure 1:**
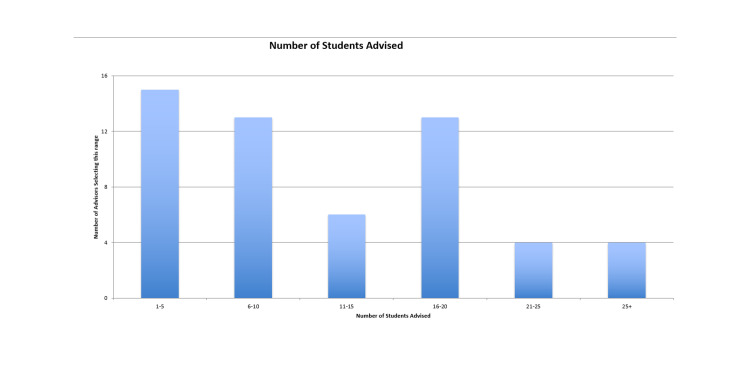
Number of students advised.

There were 56 respondents who self-identified as: 18 Program Directors (PDs), 15 Assistant and Associate Program Directors (APDs), 12 Clerkship Directors (CDs), three EM Department Chairs or Vice-Chairs, a program manager, and a director of education. Nearly all (89%) of respondents reported personally providing advice to students regarding the SVI, with four respondents stating that no institutional advice was offered to students, and two reportedly unsure. Thirty-four (61%) respondents indicated that they were the primary source of advice for the SVI for their students. CDs and assistant directors (22), along with Program Directors and Assistant/Associate PDs (22) most commonly were the source of SVI advice (79%), with residency leadership and medical school dean’s offices contributing to a lesser extent. The number of students advised per respondent ranged from one to greater than 26 students (Figure 1).

The source of advice cited by respondents was commonly consensus opinion from national organizations, including the AAMC, the Emergency Medicine Residents Association (EMRA), and CORD EM, as well as personal experience and perspective. Many different resources were allocated and utilized to advise applicants. It was common for applicants to be offered space (41%) or technical support for the interview (47%). Eight individuals stated that their institution used resources and advisors that were not specific to EM, such as the Dean’s office, and the director of speech communications, to prepare students for the SVI.

The reported time committed per faculty respondent specifically for the SVI ranged from zero to 20 hours. Associated costs of preparation included faculty time (various values attributed, including values of zero) up to $10,000 (time plus resources). Two individuals reported recommending commercial preparation resources to students. Unique resources reportedly recommended included a bootcamp dedicated to SVI preparation and a custom-made video, while at least one respondent stated that the sum of their advice was “to not worry about the SVI at all.”

Despite the wide range of preparation time and modalities, a majority (55%) of responding advisors felt that the time their students spent preparing for the SVI was just right; only one individual felt preparation time spent was too much.

## Discussion

As expected with a novel, untested, and high-stakes assessment, the number and types of resources utilized for SVI advice varied greatly, as did the estimated cost. Our data showed that some faculty members listed their SVI advising involvement as previously uncompensated time. The considerable amount of time spent advising students surely comes with some attributable cost and is not likely truly volunteered, as it is either additional work, or subtracted from other academic responsibilities, furthering the argument that the SVI served as an unfunded mandate on faculty time [11]. 

As with the unanticipated costs on faculty time, the cost to students of such mandated changes is difficult to ascertain. As noted above, some faculty recommended commercial preparation, with one resource estimating the potential cost of such a service to be $497-$2997 [12]. 

Our study found that students were commonly advised to use multimodal resources to prepare for the SVI including lists of interview questions, self and peer reflection, as well as video and practice interviews, often using institutionally provided equipment and/or space. It is important to note that the advice offered to students was largely undertaken without clear guidance on the rubric used for scoring of the SVI, and without evidence that such preparations improved scores, or that better scores would lead to increased success in the match.

Though the SVI has been discontinued, the residency video interview process was again brought to the forefront during the COVID-19 pandemic, highlighting the need to focus attention on student advising and behavioral patterns. Which resources, if any, are necessary for preparation for virtual interviews is a future area of study, as those costs ought to be balanced against any savings created by a transition to video-only interviews in the future. 

Based on the AAMC website recommendation for preparation, it appears the initial plan was for limited, self-directed preparation by the student [5]. It is evident based on our study that programs and faculty were involved in the preparation process. For any future changes to the residency application process, well-publicized guidelines for schools and advisors might help to ensure consistency and to allow schools to allocate resources appropriately.

Study limitations included the relatively low response rate, which might limit the interpretation and generalizability of the data, and the lack of other demographic data (region of respondents) that might have allowed us to identify potential skew. However, the qualitative elements of this survey allowed for exploratory data gathering in this as-yet-unexamined arena.

## Conclusions

Our study qualitatively demonstrates that the range of advice and resources used for advising the SVI was broad, and highlights additional costs of faculty time that may not have been anticipated at the onset of the operational pilot by the AAMC. Current practices and future changes to the residency application process, including the adoption of virtual interviewing formats necessitated by the COVID-19 pandemic and the piloting of new supplemental applications such as the one recently announced by AAMC for the 2021 application cycle, should include careful consideration of the unintended costs and consequences incurred and their impact on faculty, students, and institutions.

## Appendices

SVI Survey Questionnaire

1. Identify your role in advising medical students (list all applicable roles (EM clerkship director, EM residency roles, medical school advisors, etc.):

(narrative response: list title/role)

2. Do you or others affiliated with your school, program or department provide advice to students regarding SVI preparation? (Select all appropriate)

Yes, I provide advice

Yes, others provide advice

(Narrative for who gives advice)

No advice is offered

Not sure

3. Are you the primary resource for advice regarding SVI preparation at your institution?

yes/no

4. Approximately how many students did you advise regarding the Standardized Video Interview last year?

List Number:

5. Approximately how many students did you advise regarding the Standardized Video Interview THIS year?

List Number:

6. What resources are you using to inform your advice?

(narrative answer)

7. What resources are you advising students to use in order to prepare for the SVI?

Students are supplied with sample interview questions

Students practice interviews in person with an advisor

Student self-review of practice SVI video interviews

Peer review of practice SVI video interviews

Student advisor review of practice SVI video interviews

Advisor interview feedback using an advisor-developed rubric

Other (narrative answer)

8. Do you offer or recommend that there is an advisor in the room during the actual completion of the SVI? If yes, then who?

yes/no

Comment:

9. Does your medical school or residency provide physical spaces for SVI completion?

yes/no

Comment:

10. Does your medical school or residency provide computer/technical resources for SVI completion?

yes/no

Comment: 

11. Does your medical school or residency program recommend hiring outside resources for SVI preparation? 

yes/no

Comment:

12. Does your medical school or residency program use a commercial resource for SVI preparation?  If yes, please describe the type of resource.

yes/no

Comment:

13. Does your medical school or residency program use an internal institutional resource other than an advisor/EM faculty for SVI preparation?  If yes, then who?

yes/no

Comment: 

14. Please estimate how much money does your program, department, or school spends on SVI preparation services (This may include estimates of the monetary value of time spent advising, resource preparation, or the purchasing of resources for students to use).

(narrative answer)

15. Do you think your students’ preparation for the SVI is:

Too much 

Just right 

Not enough
